# Extracellular Vesicles: An Overlooked Secretion System in Cyanobacteria

**DOI:** 10.3390/life10080129

**Published:** 2020-07-31

**Authors:** Steeve Lima, Jorge Matinha-Cardoso, Paula Tamagnini, Paulo Oliveira

**Affiliations:** 1 i3S—Instituto de Investigação e Inovação em Saúde, Universidade do Porto, R. Alfredo Allen, 208, 4200-135 Porto, Portugal; steeve.lima@i3s.up.pt (S.L.); jorge.cardoso@i3s.up.pt (J.M.-C.); pmtamagn@ibmc.up.pt (P.T.); 2 IBMC—Instituto de Biologia Molecular e Celular, Universidade do Porto, R. Alfredo Allen, 208, 4200-135 Porto, Portugal; 3 Departamento de Biologia, Faculdade de Ciências, Universidade do Porto, Rua do Campo Alegre, Edifício FC4, 4169-007 Porto, Portugal

**Keywords:** cyanobacteria, extracellular vesicles, composition, biogenesis, biological roles, applications

## Abstract

In bacteria, the active transport of material from the interior to the exterior of the cell, or secretion, represents a very important mechanism of adaptation to the surrounding environment. The secretion of various types of biomolecules is mediated by a series of multiprotein complexes that cross the bacterial membrane(s), each complex dedicated to the secretion of specific substrates. In addition, biological material may also be released from the bacterial cell in the form of vesicles. Extracellular vesicles (EVs) are bilayered, nanoscale structures, derived from the bacterial cell envelope, which contain membrane components as well as soluble products. In cyanobacteria, the knowledge regarding EVs is lagging far behind compared to what is known about, for example, other Gram-negative bacteria. Here, we present a summary of the most important findings regarding EVs in Gram-negative bacteria, discussing aspects of their composition, formation processes and biological roles, and highlighting a number of technological applications tested. This lays the groundwork to raise awareness that the release of EVs by cyanobacteria likely represents an important, and yet highly disregarded, survival strategy. Furthermore, we hope to motivate future studies that can further elucidate the role of EVs in cyanobacterial cell biology and physiology.

## 1. Introduction

The phylum Cyanobacteria consists of a group of prokaryotes that typically carry out oxygenic photosynthesis with water as an electron donor and use carbon dioxide as a carbon source, or those secondarily evolved from such organisms [1]. Cyanobacteria are generally regarded as Gram-negative bacteria that can be found in a wide range of habitats [2], from aquatic to land-based ecosystems. In addition, these microorganisms can also be found in the harshest environments, such as hot springs, polar lakes, deserts and even polluted wastelands [3]. To colonize and thrive in such places, cyanobacteria evolved niche-specific adaptation strategies. They exhibit a great tolerance to environmental stresses, such as desiccation, radiation, nutrient deficiency and exposure to heavy metals [3]. Cyanobacteria adjust to these life threats through a series of adaptive physiological responses, resulting from the balance between competition for resources and intra- and interspecies cooperation [4]. This vital game of transmitting and receiving signals enable them to activate survival pathways and to maintain homeostasis. In this context, the extracellular milieu plays an important role, as it is a source of nutrients and structural components, but also of cell-damaging agents. Additionally, it can work as a sink for compounds, a channel for exchanging information, and a platform for interaction and for extending cellular functionalities [5].

**Figure 1:**
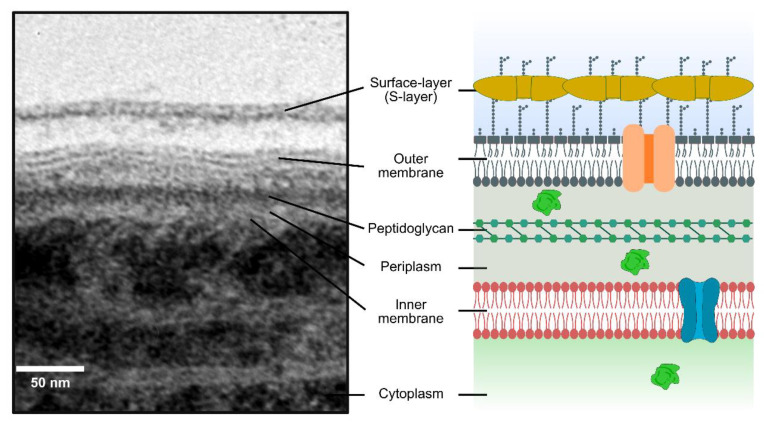
Cell envelope of the model, unicellular cyanobacterium *Synechocystis* sp. PCC 6803. Electron micrograph showing details of the *Synechocystis* sp. PCC 6803 cell envelope (left panel), which is schematically represented on the panel on the right-hand side (not to scale).

In cyanobacteria, several uptake and secretion systems have been described, which mediate the transport of various substrates. However, the knowledge regarding some of the most fundamental aspects underlying transport across the cyanobacterial cell envelope remain unknown (details of the cyanobacterial cell envelope are shown in Figure 1). For example, only recently has the outer-membrane permeability of the unicellular cyanobacterium *Synechocystis* sp. PCC 6803 been investigated [6]. Remarkably, the permeability of this cyanobacterium outer membrane was found to be 20-fold lower than that of *Escherichia coli* [6], highlighting once more that what is known for the most well-documented Gram-negative bacteria should not be directly extrapolated to cyanobacteria.

**Figure 2:**
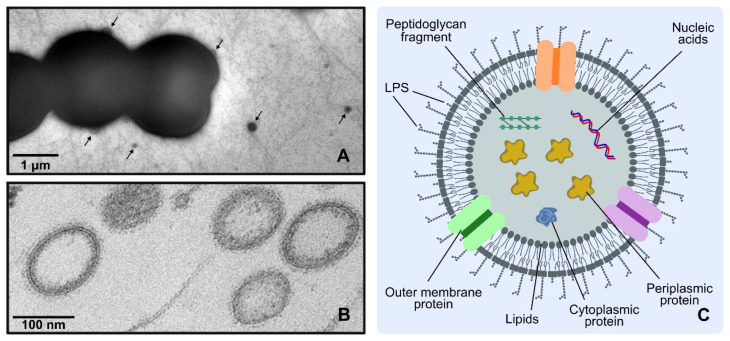
Cyanobacterial extracellular vesicles (EVs). (**A**) Transmission electron micrograph of negatively stained *Synechocystis* sp. PCC 6803 cells showing EVs (highlighted by arrows) on the cells’ surfaces and in the extracellular medium. (**B**) Transmission electron micrograph showing ultrastructural details of EVs isolated from *Synechocystis* sp. PCC 6803 culture medium. (**C**) Diagram showing the general composition of EVs from Gram-negative bacteria, including those of the marine cyanobacterium *Prochlorococcus* MED4 [31]. These vesicles are composed mainly of outer-membrane components (lipopolysaccharides (LPS); outer-membrane proteins; and lipids), periplasmic content (proteins; peptidoglycan fragments; metabolites), and, in some instances, even cytoplasmic proteins and nucleic acids (DNA and/or RNA).

A number of biomolecules have been shown to be produced and secreted by cyanobacterial cells, including proteins (e.g., [7,8,9,10]), polysaccharides (e.g., [11,12,13]), glycolipids (e.g., [14,15,16]), and even fatty acids (e.g., [17,18,19]). Moreover, secretion is also vital to rid the cyanobacterial cell from harmful, exogenous compounds that reach the cell’s interior, such as antibiotics (e.g., [9,20,21]) or excess of metals (e.g., [22,23]). Thus, product export represents an important survival strategy, playing crucial roles in a number of aspects of cyanobacterial physiology, like motility [24], adhesion [25], protection against desiccation [26], detoxification of extracellular reactive oxygen species [8,27], noxious compound efflux [9,20,21,28], etc. Throughout the years, several reports have focused on the identification and characterization of molecular machines mediating the translocation of products of metabolism, and others across the cyanobacterial inner and outer membranes (for reviews, see [29,30]). These systems, in which substrates are transported to the extracellular space via secretory portals located at the cell’s inner and outer membranes, are classified as “classical secretion systems”. Nevertheless, in terms of secretion strategies, one system has been highly overlooked in cyanobacteria—the release of extracellular vesicles (EVs), a mechanism of “non-classical secretion” (Figure 2).

EVs have been detected in a number of cyanobacterial strains, including the unicellular *Synechococcus* sp. PCC 7002 [32], *Prochlorococcus* spp., *Synechococcus* sp. WH8102 [31], *Synechocystis* sp. PCC 6803 [9,33], *Synechococcus elongatus* PCC 7942 [34] and *Cyanothece* sp. CCY 0110 [35], in the filamentous, non-heterocystous *Jaaginema litorale* LEGE 07176 [36], and in the filamentous, heterocystous *Anabaena* sp. PCC 7120 [8], *Cylindrospermopsis raciborskii* (CYRF-01) [37] and the *Azolla microphylla* symbiont [38]. However, the study of EVs in cyanobacteria has not received as much attention as in other bacteria. In this work, we focus on EVs released by Gram-negative bacteria, highlighting several aspects related to their composition, biogenesis and, most importantly, proposed biological roles. In addition, we gather the limited and fragmented information available on cyanobacterial EVs, and discuss this in light of what has been reported for other bacteria. As the biotechnological potential of cyanobacteria is becoming increasingly explored and established, we also draw special attention to several applications that have been tested and implemented with bacterial EVs. Altogether, our hopes are that researchers studying cyanobacteria become more acquainted with EVs, and that they decide to embark on the journey of elucidating further how relevant this unconventional secretion mechanism is for cyanobacterial cell survival.

## 2. EVs in Gram-Negative Bacteria

EVs were first reported more than 50 years ago [39]. Initially, they were regarded as mere artefacts of the electron microscopy technique or byproducts of bacterial growth [40]. However, subsequent studies definitively showed their production by different microorganisms [41]. Today, practically every microbial species tested, ranging from fungi to bacteria and archaea, has been shown to produce EVs, which suggests that EVs are a feature of every living cell [42]. EVs are discrete and non-replicable proteoliposomal nanoparticles [43], ranging in size between 20 and 500 nm in diameter [44]. Released by the bacterial cell envelope, EVs from Gram-negative bacteria naturally contain lipopolysaccharides, phospholipids and outer-membrane proteins; nevertheless, as EVs are not just small vesicles derived entirely from the outer membrane, periplasmic proteins, peptidoglycan fragments, and even cytoplasmic proteins and nucleic acids have been identified therein [40]. Due to the EVs’ structure and composition, their content is strongly shielded against the activity of lytic enzymes present in the environment [45], preventing cargo degradation after leaving the cell. This way, the content of EVs can reach far longer distances than biomolecules released by the classical secretion systems. Moreover, EVs contain more than one cargo, all of which are simultaneously delivered to otherwise inaccessible targets. EVs’ lumen, which resembles that of the periplasm in terms of its composition, contributes to the conservation of the cargos’ features [45]. In particular, hydrophobic compounds, or those that demand targeted deliverance, can be transported in such nanostructures. Importantly, just as in the classical secretion systems, the formation and release of EVs can be tightly regulated by the cell, and so confer an adaptive advantage under stressful conditions.

## 3. EVs’ Composition

One of the most well-characterized features of EVs is, perhaps, their biochemical composition. Several technical solutions are available to identify and quantify proteins, lipids and genetic material, which, combined with an ever increasing array of isolation methods (ultracentrifugation (including density gradient), ultrafiltration, hydrostatic dialysis, gel filtration (size-exclusion chromatography), precipitation, or even microfluidic devices and immunoprecipitation) [46], have helped clarifying many aspects of EVs’ composition and biology. As briefly presented above, and despite cell envelope particularities found among Gram-negative bacteria, EVs are generally enriched in outer-membrane components [40]. Other cellular constituents have also been detected in EVs, namely periplasmic proteins and peptidoglycan fragments [47], and also metabolites, cytoplasmic proteins and nucleic acids [40,48] (Figure 2).

Increasing evidence supports the belief that bacteria modulate EVs’ composition. For example, in *Salmonella* sp., EVs isolated from cultures grown under different conditions showed different compositions [49]. While proteins involved in translation and cellular metabolism were preferentially detected in EVs recovered from cultures grown with nutrient replete media, membrane proteins involved in nutrient acquisition were identified in EVs isolated from cultures maintained under limited nutritional conditions [49]. This study highlights the fact that EVs’ content may vary according to the physiological state of the cell, and suggests that EVs’ composition mirrors that of the parental cell at the moment of release. However, several others reported that the bacterial cell is capable of sorting what goes into EVs. The enrichment of a specific cargo that is scarce in the cell is illustrative of this fact. Conversely, the absence in EVs of highly abundant molecules in the cell and/or cell surface also indicate an EV-regulated packaging mechanism. In many bacterial species, whole-cell or outer-membrane and periplasmic proteome analyses showed differences when compared to isolated EVs [47,50]. An excellent showcase can be found in the bacteria of the genus *Bacteroides* [51,52,53]. These bacteria play a crucial role in decomposing complex polysaccharides in the human gut, being a typical resident of the intestinal microbiome. The ability is conferred by the expression of genes located in polysaccharide utilization loci (or PULs). A PUL represents a single genomic locus, which encodes proteins required to bind a specific polysaccharide at the cell surface, to perform an initial cleavage to oligosaccharides, to import these oligosaccharides into the periplasmic space, to complete the degradation into monosaccharides, and to regulate the PUL gene expression [54]. Comparative analyses between EVs and outer-membrane proteomes performed in *Bacteroides fragilis* and *B. thetaiotaomicron* revealed proteins exclusively detected either in EVs or in outer-membrane fractions [51,53]. Furthermore, EV proteomes were rich in PUL-encoded proteins, namely acidic lipoproteins with hydrolytic or carbohydrate-binding activities [51,53]. Thus, it appears that a selective packaging mechanism exists to load PUL-encoded hydrolytic enzymes into EVs [52]. Moreover, the regulated encapsulation of cargos into EVs is commonly observed in the context of pathogenesis, in which EVs work as a vehicle for virulence factors. Within the several cases reported, one highlight is the human oral *Porphyromonas gingivalis* that secretes EVs preferentially loaded with gingipains, a family of proteases that degrade host cytokines, leading to a decrease in the inflammatory response and facilitating bacterial invasion [55]. A three- to fivefold enrichment of gingipains was observed in EVs derived from *P. gingivalis* 33277 and W83, respectively, compared with levels in their parent bacterial strains [56], while major outer-membrane proteins seem to be excluded [56,57].

Because of the EVs’ composition modulation capacity demonstrated by bacteria, the population of EVs isolated from a given bacterium at a specific time point can be highly heterogeneous. To illustrate this fact, one should note that within a determined population of recovered EVs, not all the vesicles are equal regarding their physical properties, namely in terms of their size and buoyant density. Thus, when studies report the detection of certain molecules in isolated EVs, it does not mean that all vesicles contain the same types of molecules. In a study that focused on a biological comparison of EVs from *E. coli*, the authors could separate EV subgroups from crude EV preparations [58]. The isolated EV subgroups presented molecularly distinct characteristics in terms of protein and endotoxin content, and amount of RNA [58], illustrating the natural biological heterogeneity of bacterial EVs.

### Composition of Cyanobacterial EVs

Very limited information is available regarding the composition of cyanobacterial EVs. What is known is that EVs isolated from the marine cyanobacterium *Prochlorococcus* sp. have been shown to contain lipopolysaccharides, a number of typical cyanobacterial lipids (namely monoglycosyldiacylglycerol and sulfoquinovosyldiacylglycerol), and a diverse set of proteins, including periplasmic and membrane proteins (nutrient transporters, proteases, porins, hydrolases), but also cytoplasmic proteins (ribosome-associated proteins, and even RuBisCO) [31]. *Prochlorococcus* MED4 EVs were also reported to contain DNA and RNA: interestingly, DNA fragments detected in EVs measured at least 3000 bp, potentially encoding several genes. In addition, within the whole population of DNA fragments identified, an overabundance of reads was found, corresponding to a specific region of the chromosome [31]. As for RNA, sequences from as many as 95% of all open reading frames in the genome could be detected [31].

Altogether, the budding and detachment of EVs from the bacterial cell during active growth seem to constitute a regulated process rather than an accidental cell envelope disorganization event or a simple byproduct of cell lysis [40]. EVs’ composition varies in agreement with whole-cell proteomic and lipidomic changes and adaptations, or as a result of specific sorting mechanisms, both of which are intimately dependent on how EVs are formed and released, i.e., on their biogenesis. While the presence of periplasmic and outer-membrane components in EVs can be easily perceived, the detection of cytoplasmic proteins and nucleic acids require an in-depth analysis of alternative routes for vesicle production. In the following section, we focus on the EVs’ formation mechanisms.

## 4. EVs’ Biogenesis

**Figure 3:**
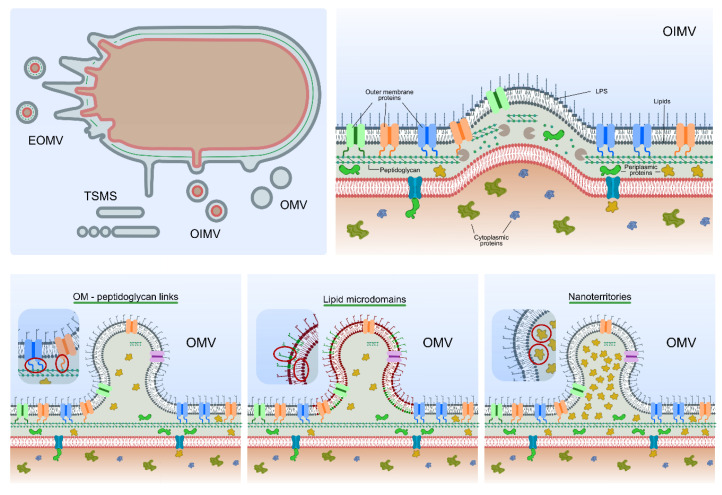
Gram-negative extracellular vesicles biogenesis models. The term “extracellular vesicle” is broadly used to describe various type of vesicles and structures released by bacterial cells. Four sub-groups of EVs have been described [41]: explosive outer-membrane vesicles (EOMV); tube-shaped membranous structures (TSMSs); outer–inner-membrane vesicles (OIMV), which have been proposed to form as a result of the action of peptidoglycan hydrolases in specific regions of the cell envelope, promoting the protrusion of the inner membrane into the periplasm and forcing the outer membrane to bulge [41,59,60]; and the classical outer-membrane vesicles (OMV) (lower panels). Different hypotheses have been formulated to explain the formation of OMVs [40,48,59], including modulation of the outer membrane–peptidoglycan links (left panel), lipid/LPS differential composition in specific regions of the outer membrane, termed lipid microdomains (middle panel), and accumulation of misfolded periplasmic proteins or envelope components in nanoterritories (right panel). Inset in each panel highlights different key components putatively involved in OMV formation, as per the respective model.

When addressing EVs’ biogenesis, a point that requires clarification is their nomenclature. In the beginning, when EVs were first described, as the vesiculation process appeared to occur exclusively from the outer membrane, EVs were designated outer-membrane vesicles (OMVs). Even today, a great majority of the published articles refer to the vesicles isolated from the extracellular milieu as OMVs. Nevertheless, recent studies have shown additional routes for vesicle formation. Accordingly, the new types of vesicles were named outer–inner-membrane vesicles (OIMVs), explosive outer-membrane vesicles (EOMVs) and tube-shaped membranous structures (TSMSs) [41]. Hence, the term extracellular vesicles (EVs) is used here to refer to the whole population of vesicles released by cells, irrespective of their origin. However, it should be noted that Gram-negative EV studies predominantly discuss OMVs, and so most of the models describing vesicle formation and release address the classic concept of OMVs [59]. Here, we try to present a general overview of the various biogenesis hypotheses (Figure 3).

One aspect that is likely common for any EV formation model is the absence of linkages between the outer membrane and the underlying peptidoglycan in the blebbing region [61]. Envelope stability in most Gram-negative bacteria results from a number of interactions between the peptidoglycan and different components located in the outer and inner membranes [40]. The peptidoglycan-interacting outer-membrane components include the lipoprotein Braun (Lpp), the outer-membrane protein A (OmpA, which is a porin), and the Tol–Pal complex [40]. Thus, areas in the envelope devoid or depleted of attachments may represent hotspots for blebbing and EV formation. Accordingly, the overall number of Lpp–peptidoglycan crosslinks has been shown in some instances to inversely correlate with EV production. In *E. coli*, NlpI is an outer-membrane protein with a role in cell division, but it has also been shown to regulate the activity of a peptidoglycan endopeptidase (Spr, or MepS) that attacks peptide bonds in the peptidoglycan, modulating its crosslinks [62,63,64]. The *nlpI* mutant is a hypervesiculating strain, presenting approximately 40% less Lpp–peptidoglycan crosslinking than the wild-type strain [63]. Conversely, the loss of the diaminopimelic acid (a component of the peptidoglycan; DAP)–DAP peptide crosslinks resulted in an increase in Lpp–peptidoglycan crosslinking levels, which, in turn, led to hypovesiculation [65]. Furthermore, mutants lacking OmpA in several genera of Gram-negative bacteria resulted in EV overproduction, likely as a consequence of reduced outer membrane–peptidoglycan crosslinking [66,67,68]. Whether or not a specific mechanism exists to modulate these linkages remains unknown [61]. Consistent with the hypothesis of the outer membrane’s dissociation from the underlying peptidoglycan, without compromising envelope integrity, portions of the peptidoglycan may be locally weakened by hydrolases in a targeted manner, allowing the invasion of the inner membrane into the periplasmic space. This protrusion, initiated by the inner membrane, ends up blebbing as an OIMV, whose envelope is similar to that of a cell. This model puts forward a possible explanation for the presence of nucleic acids and cytoplasmic proteins in EVs, as the OIMVs’ lumen originates from the cytoplasm [41,60]. Nevertheless, it is not completely certain that these OIMVs are the only EVs to contain nucleic acids, and the mechanism that enables DNA fragments to be generated and incorporated therein is far from understood.

Despite the crucial role of outer-membrane components–peptidoglycan interactions in modulating EV formation, vesicle production has also been shown to occur independently of the total level of interactions and crosslinks. In fact, mutants with high “periplasmic pressure”, resulting from an increase in misfolded periplasmic proteins, or accumulating high concentrations of envelope proteins, peptidoglycan fragments or LPS, were found to hypervesiculate [65]. This suggests an alternative route of EV biogenesis, in which these envelope components accumulate in nanoterritories, forcing the outer membrane to bulge outwards and to form a vesicle, relieving the cell from unwanted envelope components [40,59]. However, this model relies on a reduction in the crosslinking level in very specific regions, while the level of outer-membrane components–peptidoglycan crosslinks throughout the cell envelope remains constant [40,59,65].

As bacterial EVs are structurally composed of various lipid species and LPS (with rather different biophysical properties in terms of size, charge, and rigidity), determining membrane curvature and fluidity, some suggest their direct involvement in EV biogenesis. Thus, regions of the outer membrane may be enriched in particular types of lipids, LPS and/or specific LPS-associated molecules, representing lipid microdomains [40,59]. A combination of these lipid species and associated molecules may significantly increase the chance of producing outer-membrane blebs, resulting in EV production. To illustrate the role of lipid/LPS-associated molecules in the process of EVs biogenesis, one can highlight the major breakthroughs achieved in the study of the *Pseudomonas* quinolone signal (PQS). The PQS has been studied primarily in the context of its role as a quorum-sensing signaling molecule [69]. However, the exogenous addition of the hydrophobic PQS was found to affect the curvature of the outer membrane in *Pseudomonas* spp. as well as in other bacteria [70,71,72], promoting EV formation, as it incorporates into and/or fuses with LPS aggregates [70,71,72] and interacts with phospholipids [71].

Specific cellular responses may also lead to EV formation, which is particularly important in the context of environmental settings. This is the case for EOMVs, which are EVs formed as a consequence of cell lysis, triggered by phage-derived endolysins that degrade the peptidoglycan, after which the cell rounds up and explodes [41,73,74]. This mechanism of EV release has only been observed and reported in *Pseudomonas aeruginosa* [74], but is likely to occur in many other Gram-negative bacteria. EOMVs are formed due to the normal tendency of shattered membrane fragments to reorganize into vesicles, randomly capturing buoyant cellular material [41]. Hence, EOMVs’ lumenal content may vary significantly in comparison to other EVs.

Finally, TSMSs represent a specialized type of EVs, comprising nanotubes, nanowires and nanopods. These structures are tube-like protrusions of the outer membrane, forming bridges between cells at the level of the periplasmic space, particularly produced in biofilms [41] but also detected in liquid media [75]. It has been suggested that TSMSs may facilitate the transfer between the cells of periplasmic proteins and metabolites, membrane proteins, and lipids [41,59]. Not much is known about how these structures are formed, and the simplest version detected was a chain of EVs in *Myxococcus xanthus* [76]. However, EVs have also been observed to pinch off from nanotubes in *Vibrio vulnificus* [77].

The models presented above elegantly address the contribution of various factors in EVs’ biogenesis, and no vesicle formation process proposed thus far is widely accepted as the consensus. As the hypotheses discussed are not mutually exclusive, it is possible that a combination of factors and mechanisms operate to produce vesicles. Still, in the field of EV biogenesis, and in light of the presented models, a challenge that remains largely unsolved is to explain how soluble cargo is selected for EV packaging. It has been proposed that the sorting of soluble bacterial proteins into EVs may result from direct or indirect interactions with the periplasmic face of specific integral or auxiliary outer-membrane components, or even lipids, which are likely to be included in future vesicles. Conversely, components may be excluded from EVs by similar interactions with outer-membrane components that are not prone to budding [40]. Nevertheless, more experimental data are needed to validate this model.

### EVs’ Formation in Cyanobacteria

Several electron micrographs have shown EVs on the cell wall of different cyanobacterial strains (Figure 2; e.g., [9,31,36,78]). Nevertheless, the knowledge regarding EVs’ biogenesis in these microorganisms is still very limited. Owing to the unique features presented by the cyanobacterial cell envelope, it can be anticipated that cyanobacterial EVs may have a distinct composition and rather specific formation mechanisms. On the outer-membrane level, structural differences in the lipid A moiety of LPS compared to that usually found in enterobacteria (in most cyanobacteria, the glucosamine disaccharide backbone is not phosphorylated, and the hydroxyl fatty acid chains are different [30]), the presence of species-specific carotenoids [79], the absence of the Braun lipoprotein Lpp (which establishes covalent interactions with the peptidoglycan; see above), are just a few of the factors that may determine specific mechanistic adaptations for EVs’ biogenesis in cyanobacteria. Others may include peptidoglycan thickness (significantly higher when compared to other Gram-negative bacteria), the extent of peptidoglycan crosslinking (close to 60%, as opposed to the usual 20–30% found in most Gram-negative bacteria), the presence of specific polysaccharides complexed with the peptidoglycan [80], and unique lipid composition (while most bacteria contain phospholipids as major glycerolipids, cyanobacteria generally contain three glycolipids, monogalactosyldiacylglycerol, digalactosyldiacylglycerol and sulfoquinovosyldiacylglycerol, and a phospholipid, phosphatidylglycerol [81]). In addition, some cyanobacterial strains possess a surface layer (S-layer) (see Figure 1), the outermost structure of the cell envelope, composed of a glycoprotein with the capacity of self-assembly [82]. The S-layer in *Synechocystis* sp. PCC 6803 has been proposed to contribute to the integrity of the cell wall [83], conferring mechanical and osmotic cell stabilization, almost as an exoskeleton [84]. Using a number of *Synechocystis* sp. PCC 6803 mutant strains, impaired in several secretory functions, Gonçalves et al. observed that strains lacking the S-layer consistently presented a higher vesiculation capacity [28]. The authors hypothesized that the S-layer in *Synechocystis* sp. PCC 6803 represents a physical barrier for the biogenesis and release of EVs [28].

In summary, Gram-negative bacteria produce different types of EVs (OMVs, OIMVs, EOMVs and TSMSs). Remarkably, the production of EVs is apparently a vital mechanism as neither a bacterial species nor a mutant without the capacity to release EVs has been reported thus far [42]. As EVs’ composition is intimately determined by the biogenesis process, an in-depth understanding of these mechanisms will continuously contribute to appreciate how bacteria selectively package EVs, and uncover their biological roles.

## 5. EVs’ Biological Roles

**Figure 4:**
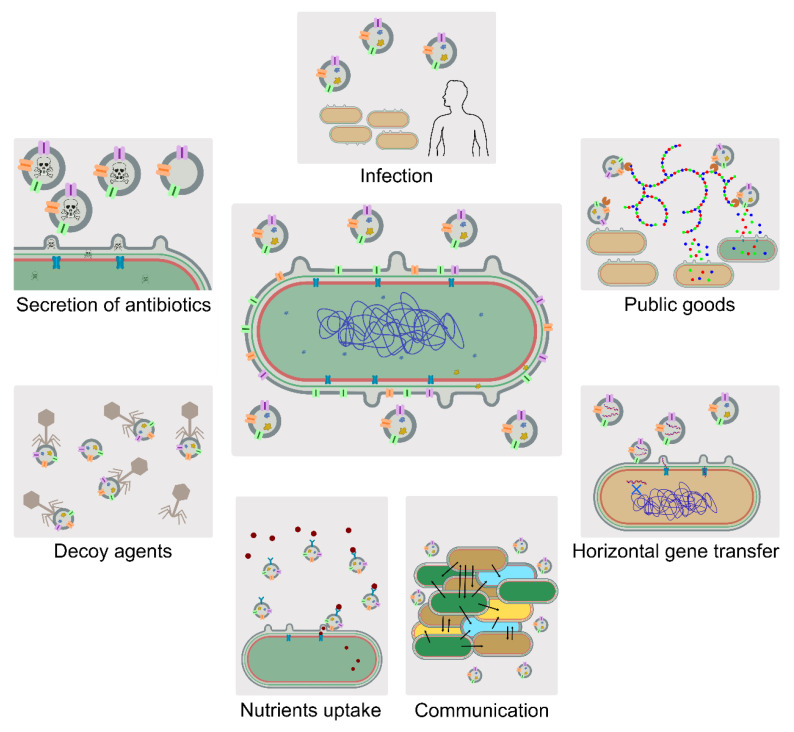
Diagram summarizing the biological roles proposed for extracellular vesicles from Gram-negative bacteria. EVs may have the following functions: in infection, by eluding the host immune response; as public goods, as they may carry catalytic enzymes that help deconstructing complex extracellular biomolecules, making them available for the EVs-releasing bacteria and for other organisms; in horizontal gene transfer, as they transport nucleic acids that can be transferred between organisms; in communication, e.g., in the context of biofilms; in the uptake of nutrients (e.g., [96,97]); as decoy, for example to evade phage infection; and in the secretion of antibiotics (e.g., [98,99]).

Today, bacterial EVs are well established and are accepted to be involved in several biological functions. Intra- and interspecies communication, defense, the uptake of resources, the release of metabolic byproducts, detoxification, responsiveness to abrupt envelope stresses, horizontal gene transfer, acting as ‘decoy’ agents, as well as functioning as public goods, are among the proposed roles of bacterial EVs [40,48] (Figure 4). For instance, *Helicobacter pylori* releases EVs selectively decorated with catalase KatA on their surface, which contributes to neutralizing reactive oxygen species from the host, working as a mechanism to aid in evading host immune responses during infection [85]. Interestingly, *H. pylori* EVs showed a better performance in hydrogen peroxide detoxification than whole-cell lysates [85]. Moreover, Lekmeechai et al. demonstrated that these KatA-decorated EVs were able to protect *H. pylori katA*-null mutants from the hydrogen peroxide noxious effects, while EVs from these mutants or heat-inactivated KatA-decorated EVs could not [85]. With this example, it is possible to highlight that: (i) EVs can be enriched with a crucial biological component, which is active in the extracellular environment; (ii) EVs contribute to host infection; and (iii) EVs released by specific bacteria may aid other bacteria to promote survival. Moreover, there are numerous cases in which antibiotic resistance enzymes have been detected in isolated EVs [86,87,88], resulting not only in a defense strategy, but also in large-spectrum community tactics. In this context, nucleic acid exchange between bacterial cells via EVs [89,90,91] represents a serious health-threatening issue, as antibiotic resistance determinants encoded in genetic material may be transferred by horizontal gene transfer. Another example to highlight EVs importance is their presence in biofilms [70,92,93], which are competitive niches that can comprise several bacterial species. It is thought that, due to the natural structure and secreting advantages of EVs, they could have a role in communication through the biofilm matrix to ease bacterial cooperation [70]. Biofilms may also function as well as a way to adhere to certain surfaces to facilitate the process of invasion, in which EVs may also play a critical role. That is the case described for EVs from the oral pathogen *P. gingivalis*, which enables the recruitment and adhesion of other bacterial players to the biofilm, increasing the chance of dental plaque formation [50,94]. The role of EVs in biofilms can be further illustrated by *Xylella fastidiosa*, a serious crop-threatening pathogen. During plant colonization, *X. fastidiosa* migrates and proliferates within xylem vessels. It has been shown that *X. fastidiosa* hypervesiculating mutants present a higher virulence capacity than the wild-type bacterium [95]. It was proposed that the production of EVs by *X. fastidiosa* represents a system that helps to control the state of biofilm formation: as the surface composition of EVs released by *X. fastidiosa* is very similar to that of the cells, EVs bind to the xylem vessels and block the attachment of *X. fastidiosa* to surfaces [95]. This allows the bacterial cell to modulate its adhesion capacity, in order to change from an “exploratory” lifestyle, for spreading within the plant host, to a more adhesive type, prone to insect transmission [95].

### Biological Functions of EVs in Cyanobacteria

Cyanobacterial EVs have also been associated with key physiological roles. For instance, an increase in vesiculation was observed in *Cylindrospermopsis raciborskii* CYRF-01 in response to UV radiation [37]. A similar hypervesiculation response was detected when this cyanobacterium was co-cultured with *Microcystis aeruginosa* (MIRF-01). The authors suggested that hypervesiculation was an adaption response to the physical and biological environmental stressors [37]. Moreover, the work by Biller and co-workers on *Prochlorococcus* sp. EVs suggested that these spherical nanostructures can work as a “smoke-screen”, preventing cyanophage infection [31]. Due to the similar composition between outer membranes from *Prochlorococcus* sp. and their EVs, cyanophage receptors are equally found on living cells and vesicles. This could explain the numerous accounts of phages bound to vesicles reported by Biller et al. [31], some of which seemed to have injected their genetic material into the EV. Thus, EVs may help the cell to escape phage infection, and so represent a crucial element of cyanobacterial population dynamics in the ocean. As *Prochlorococcus* sp. are the main primary producers in open oceans [100], this work represents also a major breakthrough in the understanding of the role of cyanobacterial EVs in other ecological aspects, namely as vectors for carbon fluxes in the ocean, as the metabolites and biomolecules packaged in *Prochlorococcus*-derived EVs were shown to be able to exclusively sustain the growth of heterotrophic bacteria [31]. Moreover, nucleic acids were found in EVs released by this highly abundant microorganism. Thus, vesicles from *Prochlorococcus* sp. truly represent a reservoir of genetic information, proposed to be active in horizontal gene transfer in marine ecosystems [31,101]. Another report supports the presence of DNA in cyanobacterial EVs: Zheng et al., showed that the cyanobiont present in the water fern *Azolla microphylla* sporocarp abundantly releases EVs [38]. Remarkably, up to 90% of the detected vesicles that were released by the cyanobiont filaments upon their initiation of proakinete/akinete differentiation contained DNA [38]. This observation suggests possible lateral gene transfer between the symbiotic partners. In addition, the released EVs may also contribute to cyanobacterial biofilm formation and outer envelope development during akinete differentiation and during sporocarp development, as proposed [38].

In line with reports from other Gram-negative bacteria, vesiculation in cyanobacteria also seems to be adjusted in response to specific genetic modifications, further suggesting that EVs may contribute to the homeostasis of the cyanobacterial cell. The deletion of the two glycogen synthase genes (*glgA*-I and *glgA*-II) in *Synechococcus* sp. PCC 7002 resulted not only in an accumulation of soluble sugars, but also in the spontaneous secretion of high levels of soluble sugars into the medium, without the need for additional transporters [32]. Moreover, numerous EVs budding from the outer membrane of the mutant could be observed, and the authors hypothesized that the release of EVs could mediate soluble sugar excretion [32]. In a different work, the deletion of the gene encoding the outer-membrane protein TolC in *Synechocystis* sp. PCC 6803 resulted in a strain with a high vesiculation capacity [9]. TolC is crucial for the export of several types of biomolecules, ranging from proteins [7,9,25,28] to fatty acids [17], and exogenous compounds that reach the cytoplasm [9,20,21,28]. It was proposed that the vesiculation was a response to the impaired classical secretion [9]. In a follow up study, deletion mutants in some inner-membrane and periplasmic components, parts of the TolC-dependent efflux pumps, showed as well-altered vesiculation [28]. Mutants with a higher vesiculation capacity than the wild-type strain were found to lack the S-layer (see above, EVs’ biogenesis). Nevertheless, none of these mutant strains reached the high vesiculation level presented by the *tolC*-deletion mutant [28]. It remains to be fully determined why the *tolC*-deletion mutant hypervesiculates, but there is evidence that the strain is under some sort of envelope stress, as specific periplasmic chaperones and proteases are highly upregulated (*spy* and *degQ*) and overexpressed (Spy and DegP) compared to the wild-type [28]. Genetic modifications in cyanobacteria that affect vesiculation do not always result in hypervesiculation. In a recent work, a hypovesiculating *Synechocystis* sp. PCC 6803 mutant was reported, in which the gene encoding the alternative sigma factor SigF was deleted [102]. Nonetheless, SigF was found to be a broad player in the control of several metabolic and cellular processes, including different secretion mechanisms. The lower vesiculation capacity was therefore proposed to be part of a large adaptation process, rather than a specific target of SigF regulation [102].

As more bacterial species become characterized in terms of their EVs production capacities, more biological roles are attributed to EVs. The fact that many of these bacteria can be genetically modified and engineered has contributed towards the recognition that bacterial EVs may be used to fulfil specific technological goals.

## 6. Biotechnological Applications of Bacterial EVs

**Figure 5:**
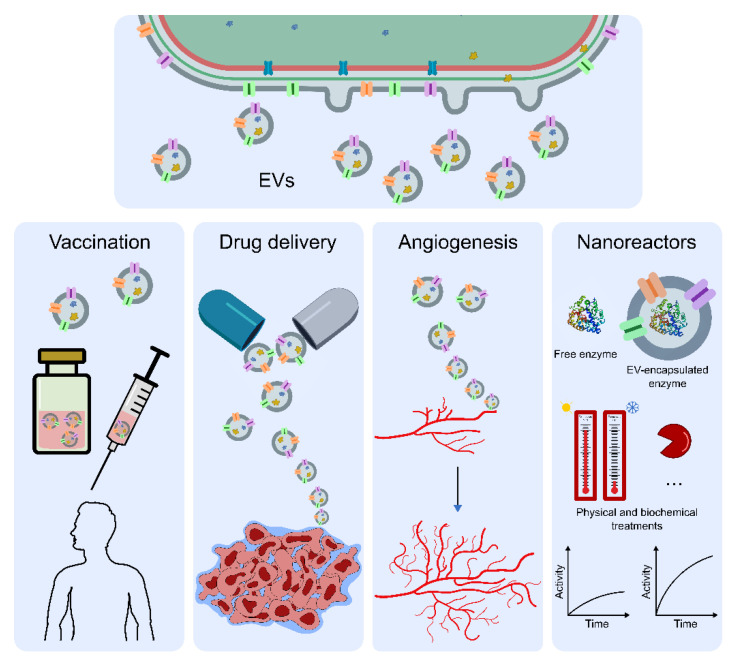
Illustration summarizing some applications that have been tested with bacterial EVs. One field that has benefited enormously from the potential of bacterial EVs is biomedicine. The best example is perhaps the use of EVs in vaccination, for which the commercial vaccine Bexsero^®^ is a case of widely accepted success. Bacterial EVs have also been tested with promising results to deliver cargo in a cell-specific manner, in particular in the context of cancer research (e.g., [105,106]). EVs from the cyanobacterium *Synechococcus elongatus* PCC 7942 have been shown to positively modulate and even promote angiogenesis in the context of wound healing. Bacteria have also been genetically engineered as to release EVs packaged with enzymes, contributing to the protection of enzymatic functions (e.g., [107]), in what the EVs may be regarded as nanoreactors.

Fundamental research carried out in the past 20–30 years has significantly contributed to our better understanding of the mechanisms of EVs biogenesis in bacteria, their composition and properties, as well as their biological roles. The capacity of EVs to package multiple cargos, the fact that cargo properties are maintained in EVs, and the extraordinary advantage of EVs in protecting and trafficking cargo to otherwise inaccessible targets [45] are just a few of the EV attributes that have promoted their application in biotechnology. Furthermore, through the constant developments in bioengineering and genetic editing techniques, it is now possible to tailor EVs’ exterior features, as well as modulate their cargo [103,104]. As a result, EV-based nanotechnology has been evolving quickly into a powerful and innovative toolkit for various fields (Figure 5), even though several challenges still need to be addressed, mainly regarding their scalability, commercial viability, and safe and efficient clinical use [103].

Inherent to the constant necessity of developing novel and more efficient vaccines against various pathogenic agents, EVs have been successfully implemented as potential vaccines/antigen-delivery platforms. Two major strategies have been followed for the development of EV-based vaccination. On one hand, naturally derived EVs from the target pathogenic bacterium can be isolated and directly administered to the subject. In this case, EV protection is mediated by the abundance of pathogen-associated molecular patterns, known to play a key role in stimulating innate immunity and promoting adaptive immune responses. The best example is the EV-containing meningococcal vaccine against *Neisseria meningitidis*, currently commercialized as Bexsero^®^ [96,108,109,110]. On the other hand, through a genetic engineering approach, recombinant EVs harboring heterologous antigen(s) can be directly obtained from a non-pathogenic bacterial model. In this way, it is possible to develop safe and standardized EVs, capable of eliciting an adequate immune response with great versatility and flexibility [111,112,113]. The use of engineered *E. coli*-derived EVs loaded with heterologous proteins to work as antigen-delivery vehicles has been tried with very promising results [111]. All of these efforts towards the development of EV-based vaccine strategies offer the opportunity to accomplish highly effective, easy to produce and multi-valent vaccines.

EVs have also been used as specialized carriers for the delivery of therapeutic compounds [103,104]. For instance, bioengineered EVs produced by *E. coli*, presenting a human epidermal growth factor receptor 2-specific affibody molecule on their surface, could target cancer cells in a mouse model in a cell-specific manner [105]. As EVs were loaded with a small interfering RNA (siRNA), the specificity of the EVs’ delivery resulted in targeted gene silencing, and thus in a reduction in the protein levels of a key enzyme involved in mitosis, ultimately inhibiting cancer cell proliferation [105]. Moreover, these nanostructures have also revealed their great potential in the delivery of chemotherapeutic agents, as demonstrated with EVs from an attenuated *Klebsiella pneumonia* strain that efficiently delivered the drug doxorubicin to lung cancer cells [106]. Moreover, in the context of cancer, EVs have also been reported as a promising tool for immunotherapy strategies, not only through EV-induced long-term antitumor immune responses [114], but also through their decoration with tumor antigens towards antigen-specific antitumor therapy [115]. Despite the fact that most efforts have been directed towards anti-cancer/anti-tumor therapies, other delivery applications have also been associated with EVs, including their development as antibiotic delivery vehicles. Recently, through the identification of a novel drug resistance mechanism mediated by EVs in *Acinetobacter baumannii*, antibiotic-loaded EVs were shown not only to efficiently deliver and facilitate the entry of antibiotics into pathogenic bacteria, but also to support the sustained bactericidal effect in the intestinal tract of a mouse model [99]. In an era of global concern regarding drug-resistant bacteria, this reported novel class of EVs mediating the delivery of antibiotics may turn out to be extremely useful in reducing antibiotic dosage, and in increasing the bactericidal effect.

Other applications are also being considered and developed, including the use of EVs as natural and versatile nanoreactors. The systematic organization of enzymes for the efficient operation of cascade reactions, resulting in cellulose degradation, has been demonstrated through the use of *E. coli*-derived EVs [116]. The functional assembly of multiple enzymes on the EV surface was accomplished by the construction of a system comprising three orthogonal cohesion domains and a cellulose-binding module, all assembled and anchored through OmpA [116]. By adding their species-specific dockerin parts, all cellulases could be activated, resulting in the amplification of cellulose hydrolysis by more than 20-fold over that of non-complexed enzymes [116]. In addition to performing complex biocatalysis, EVs have also been recently described to protect the native properties and enzymatic activity of phosphotriesterase (PTE), an ideal enzyme for bioremediation [107]. In light of this discovery, it is plausible to extend this EV-mediated enzymatic packaging/protection strategy to other biotechnological fields, including the health sciences context, for example, where the prevention of enzyme degradation on human serum would lead to a more efficient enzymatic delivery and bioactivity.

### Cyanobacterial EVs in Biotechnology

Although most of the reports available on cyanobacterial EVs are fundamentally directed towards basic research on EV biology, these microorganisms represent an untapped tool with attractive features and promising applications. Indeed, EVs from *Synechococcus elongatus* PCC 7942 have already been linked to the promotion of angiogenesis and wound healing in vitro and in vivo through the promotion of interleukin 6 expression, highlighting their potential in the field of biomedicine [34]. As novel insights are gained in respect to cyanobacterial EVs, this can be the first of a broad range of prospective applications. In fact, cyanobacterial EVs might have a significant advantage over other bacteria in relation to a relevant issue concerning EVs’ structural components, namely regarding LPS. In high dosages, LPS from some Gram-negative bacteria, including *E. coli*, *Salmonella* and *Pseudomonas*, can lead to pathological reactions, including systemic inflammatory responses [117]. Consequently, to avoid toxicity in biomedical applications, administration to the subjects of EVs derived from such organisms must follow rigorous and complex administration schemes, and/or demand vesicle bioprocessing approaches by implementing attenuated mutants or additional LPS detoxification steps [118,119]. Remarkably, evidence from different sources strongly suggests a significantly reduced toxicity in cyanobacterial LPS [120,121,122]. This low immune reactivity against cyanobacterial LPS can be explained by the missing phosphorylation at positions 1-4′ of the glucosamine disaccharide in the cyanobacterial lipid A backbone [30]. The possibility that cyanobacterial LPS do not possess true endotoxic effects that can harm human health [30] underlines the potential of cyanobacteria-derived EVs as valid and safe alternatives to overcome this LPS-induced toxicity hurdle. With the current efforts regarding the development of more robust and reliable molecular tools to metabolically engineer cyanobacteria, EVs may become the target for packaging products of interest that can range from soluble and membrane-bound enzymes, to lipids, long-chain carbon molecules, and metabolites. With this in mind, an array of interesting applications may soon be associated with cyanobacterial EVs.

## 7. Conclusions

The EV research field is not novel, but there has always been some skepticism among researchers as to the existence and physiological function of EVs [42]. Some of the frequent arguments include the possibility of vesicles being artefacts of lipid self-aggregation, or debris from lysed cells, or even just ordinary waste products. Moreover, other argue that EV release represents a waste of energy, and thus serves no purpose, or that bacteria lack the complex machinery present in eukaryotic organisms determining the release of vesicular bodies, or even, as there are no mutants lacking vesiculation capacity, that EV release cannot be a regulated process [42]. Recent technical advances to isolate and analyze EVs have contributed significantly to dismissing these doubts. As the field develops and EVs become increasingly accepted, there is a demand for standardizing EV research through increased systematic reporting, which can already be followed on the Transparent Reporting and Centralizing Knowledge in Extracellular Vesicle Research (EV-TRACK) database [123]. In addition, the International Society for Extracellular Vesicles has also published guidelines for EV research, which are frequently updated.

In cyanobacteria, research into EVs is only just emerging, but the limited reports available already suggest that cyanobacterial EVs fulfil important ecological functions, such as evading phage infection [31]. In other instances, vesiculation was detected in response to the deletion of key metabolic enzymes [32] or transporters [9,28], but the full biological meaning of such observations remains to be elucidated. Nevertheless, it is clear that vesiculation in cyanobacteria can be modulated in response to environmental cues [37], thus pointing to the strong possibility of contributing to cell homeostasis and, ultimately, adaptation and survival. The detection of EVs in the context of symbiotic associations [38] opens the door to poorly addressed inter-kingdom communication, with large implications. In particular, the detection of genetic material therein may represent a route of gene transfer, maybe accounting for the presence of cyanobacterial genes in plant nuclear genomes [124].

In the past few years, the advances in the understanding of cyanobacterial biology and metabolism have been intimately related to rapid developments in synthetic biology and metabolic engineering. In addition, these contributions have also placed cyanobacteria in the spotlight in biotechnology, garnering increasing interest from several sectors. Combining cyanobacterial genetic tractability with metabolic plasticity, cyanobacteria can easily become attractive models for the production of EVs with tailored cargo. Some reports indicate that cyanobacterial lipopolysaccharides are less toxic to mammals compared to those isolated from other bacteria [121,122], which can be relevant for EV-mediated delivery.

In summary, the study of EVs in cyanobacteria definitely deserves more attention. Several open questions related to physiological responses, cellular modifications, differentiation, communication strategies and adaptability might be re-analyzed in light of the existence of EVs. Future advances in this field will surely contribute to a better understanding of how cyanobacteria have made their way through the last 2.1 Ga of the Earth’s history, while shaping life as we know it.
